# Adiponectin Fractions Influence the Development of Posttransplant Diabetes Mellitus and Cardiovascular Disease in Japanese Renal Transplant Recipients

**DOI:** 10.1371/journal.pone.0163899

**Published:** 2016-10-05

**Authors:** Hiroki Adachi, Kanae Nakayama, Norifumi Hayashi, Yuki Matsui, Keiji Fujimoto, Hideki Yamaya, Hisao Tonami, Hitoshi Yokoyama

**Affiliations:** 1 Department of Nephrology, Kanazawa Medical University School of Medicine, Uchinada, Ishikawa, Japan; 2 Department of Radiology, Kanazawa Medical University School of Medicine, Uchinada, Ishikawa, Japan; The University of Tokyo, JAPAN

## Abstract

**Background:**

A few studies have investigated the role of adiponectin fraction for cardiovascular disease (CVD) in RTx recipients.

**Subjects and Methods:**

We studied 57 adult subjects (39 males, 18 females; 10 cadaveric donors) with at least three years of allograft survival (median 251 months). We examined clinical backgrounds such as treated drugs, blood pressure (BP, mmHg), body mass index (BMI), and blood chemistry including cholesterol (total, LDL-C, HDL-C), glucose, glycated hemoglobin (HbA1c), and serum high and low-molecular-weight (HMW/LMW) ADPN fractions with regard to the associations of the visceral and subcutaneous fat areas on CT scan. We also analyzed the associations of CVD and post-transplant diabetes (PTDM) with ADPN fractions and the fat areas.

**Results:**

The visceral fat area was inversely correlated with serum HMW and LMW ADPN levels and HMW ADPN ratio (r = -0.400, p = 0.002 and r = -0.296, p = 0.025 and r = -0.444, p<0.001, respectively). Furthermore, the visceral fat area was positively with the LMW ADPN ratio (r = 0.467, p<0.001), but no significant correlation was noted between the subcutaneous fat area and the ADPN ratio. On multiple regression analysis, eGFR and the visceral fat area were significant reducing factors of HMW ADPN levels, and the alteration of eGFR was identified as an increasing factor of HMW ADPN levels. Patients with CVD had larger visceral fat area (p = 0.004), lower HMW ADPN ratio (p = 0.022) and higher LMW ADPN ratio (p = 0.049). In addition, the higher HMW ADPN ratio and statin treatment were identified as reducing factors of the development of CVD, but the LDL-C level was an aggravating factor. Moreover, the higher LMW ADPN ratio and the visceral fat area were aggravating factors of PTDM.

**Conclusion:**

Even in Japanese renal transplant recipients, visceral fat area and ADPN fractions were significant factors for the development of both CVD and PTDM.

## Introduction

The causes of chronic renal graft failure have been roughly classified into immunological and non-immunological, with dyslipidemia, which belongs to the latter, influencing renal graft function. From the viewpoint of chronic kidney disease (CKD), complications of the cardiovascular system following kidney transplantation have a strong impact on the prognosis of renal graft recipients, for whom the factors constituting metabolic syndrome (obesity, diabetes, hypertension, and dyslipidemia) as well as transplantation-related immunosuppressors, the state of CKD, proteinuria, and anemia are also important[1, 2].

Adiponectin (ADPN) is mainly produced and subsequently secreted by the adipocytes in white and brown adipose tissues. ADPN has been reported to improve insulin sensitivity and exert anti-diabetic, anti-arteriosclerotic, and anti-inflammatory effects [3]. Reductions in ADPN plasma levels promote arteriosclerotic cardiovascular events, hypertension, and dyslipidemia[4]. However, ADPN levels are high in CKD patients, indicating that ADPN is metabolized in the kidney[5]. Martinez et al. compared end-stage renal disease patients with a normal renal function group, and suggested that ADPN mRNA and protein expression in adipose tissues is enhanced in renal failure patients due not only to renal hypofunction-induced reductions in metabolism, but also the increased production of ADPN in adipose tissues [6]. Regarding ADPN, a previous study reported that its high-molecular-weight (HMW) dodecamer and 18-mer, but not its monomer or trimer were closely associated with the prevention of coronary arterial disease, weight loss effects, and improved insulin resistance [7]. It has been clinically shown that a reduction in ADPN is a useful marker for the future development of diabetes [8], and is also a predictive marker of cardiovascular disease (CVD) [9]. However, the relationships between post-transplant diabetes mellitus (PTDM) and CVD and the ADPN fraction in renal transplant patients have not yet been examined.

Otherwise, a number of clinical epidemiological studies have reported that inflammatory markers, such as high-sensitivity CRP(C-reactive protein), tumor necrosis factor(TNF-α), and interleukin-6(IL-6), are associated with insulin resistance and visceral fat accumulation [10], and an inverse correlation has been detected between ADPN levels and the visceral fat area (VFA) [11]. However, the relationships between fat areas and the ADPN fraction, PTDM, and CVD in renal transplant patients currently remain unknown. In the present study, the serum lipid markers, including the ADPN fraction, were measured and their relationship with renal graft function was investigated to evaluate the importance of lipid control for renal graft function. VFA and the subcutaneous fat area (SFA) were also measured on CT to investigate their relationships with serum lipid markers, PTDM, and CVD. The results obtained demonstrated that serum ADPN is associated with PTDM and CVD.

## Subjects and Methods

Our subjects comprised 57 patients (39 males and 18 females) who had undergone renal transplantation at Kanazawa Medical University Hospital and in whom the transplant had been engrafted for 3 years or longer at the initiation of observations in 2004, serum creatinine levels were 6 mg/dL or lower, and engraftment persisted until 2012. The causes of renal failure in the transplant patients were chronic glomerulonephritis (n = 49), reflux nephropathy(n = 3), hypoplasia(n = 2), unknown etiology(n = 3).

Our study didn't include vulnerable populations include prisoners, subjects with reduced mental capacity due to illness or age, and children. In addition, We used only blood and radiological sample in this study. This study was approved by the Ethics Committee of Kanazawa Medical University (Kanazawa Medical University Epidemiological Study Review No. 240). All patients provided written informed consent. This study was conducted according to the principles of the Declaration of Helsinki and Istanbul.

### Clinical findings and laboratory data

The influencing factors investigated were age at transplantation, sex, living donor or cadaver renal transplant, the human leukocyte antigen (HLA) concordance rate, time after transplantation, estimated Glomerular Filtration Rate (eGFR) at the initiation of observations, body mass index (BMI), blood pressure, total cholesterol (T chol), high-density lipoprotein cholesterol (HDL-C), low-density lipoprotein cholesterol (LDL-C), non HDL-C (= T chol—HDL-C), anti-diabetic drugs, immunosuppressive drugs, statins, anti-hypertensive drugs, hypoglycemic agents including insulin, the serum ADPN fraction (HMW, middle- (MMW), and low-molecular-weight (LMW) ADPN), and VFA and SFA. The items investigated were the relationships between serum ADPN levels and eGFR and between VFA and SFA and the serum ADPN level and fraction, as well as the factors influencing the development of PTDM and CVD.

CVD was defined as cases meeting the criteria of ischemic heart or coronary artery disease, cerebrovascular disease, and diseases of the aorta and arteries proposed by the WHO.

### Measurement methods

Serum creatinine was analyzed using an enzymatic method with the Hitachi creatinine auto-analyzer model 7170 (Hitachi, Tokyo, Japan) and an enzyme solution (Preauto-SCrE-N; Daiichi Pure Chemicals Co., Tokyo, Japan). T chol, LDL-C and HDL-C were measured by direct enzymatic assays using an automatic analyzer (Hitachi, Tokyo, Japan). Serum total, HMW, MMW, and LMW ADPN were measured using a sensitive enzyme-linked immunosorbent assay kit (SEKISUI MEDICAL Co., Tokyo, Japan). Renal function was evaluated based on eGFR. eGFR (= 194 x SCr^-1.094^ x age^-0.287^ x 0.739 for females, ml/min/1.73m^2^) was calculated based on the patients’ serum Cr levels, as described previously [12].

As indices of obesity, the total fat area, SFA, and VFA of the trunk were measured setting the baselines to those in the umbilical-level cross-section in a supine position on computed tomography (CT). VFA (cm^2^) and SFA (cm^2^) were calculated from this image.

### Statistical analysis

All continuous variables were presented as the median and interquartile range. The Mann-Whitney test was used for comparisons between donor types and also the sexes. The relationships between lipid markers and the serum ADPN fraction, VFA, and SFA were evaluated using Spearman’s correlation coefficient. In order to compare serum ADPN between obese and non-obese patients, the relationships between the development of PTDM and fat areas and serum ADPN and also between CVD and fat areas and serum ADPN were evaluated using the Mann-Whitney test. The factors influencing the HMW ADPN level and its ratio were analyzed using a multiple regression analysis. The factors influencing the development of PTDM and CVD were subjected to a multiple logistic regression analysis. Stat Flex Version 6 (Artech Co., Ltd., Osaka, Japan) was used as the statistical analysis software.

## Results

### Clinical background

Our subjects comprised 57 renal transplant patients (39 males and 18 females; 47 living donors and 10 cadaveric donors, in Table 1). Systolic and diastolic blood pressures were 126 (120–132) and 76 (70–84) mmHg, respectively, being stable, with 51 subjects (89%) meeting the target blood pressure (systolic blood pressure lower than 140 mmHg). Regarding cholesterol, LDL-C was 120 mg/dL or lower in 41 (72%), while HDL-C was 40 mg/dL or higher in 56 (98%). Thirty-three subjects (58%) were being treated with pravastatin or atorvastatin. As concern to the donor types, the age at transplantation was higher in the cadaver transplant group (p<0.01), while the post-transplant follow-up period was longer in the living donor transplant group (p<0.01). No significant difference was noted in the number of HLA mismatch or eGFR, nor were there any significant differences in the serum ADPN level or fat areas. Regarding drugs, calcineurin inhibitors were frequently used in the cadaver transplant group, while oral anti-diabetic agents were frequently used in the living donor transplant group (p<0.01 and p<0.01, respectively).

**Table 1:** Characteristics of study subjects in transplants.

Variable	Total	Living	Cadaveric
N	57	47	10
Gender male: female	39:18	34:13	5:5
Age at transplantation, year	32.0(25.0–36.0)	29.0(22.8–35.0)	43.0(38.0–49.0)**
HLA mismatches, median (range)			
HLA-A/B	2(0–4)	2(0–4)	2(0–4)
HLA-DR	1(0–2)	1(0–2)	0(0–2)
Time after Tx, month	257(188–333)	266(204–344)	186(166–205)**
BMI	21.2(19.1–22.9)	21.2(19.0–23.7)	21.3(19.1–23.7)
Blood pressure, mmHg			
Systolic	126(120–132)	126(119–132)	126(124–128)
Diastolic	76(70–84)	76(70–84)	70(68–84)
eGFR at 2004 (ml/min)	55.2(43.5–68.6)	54.8(42.8–66.1)	60.2(53.5–71.7)
eGFR at 2008 (ml/min)	47.7(37.8–57.8)	44.6(38.1–57.3)	58.6(51.0–68.7)
eGFR at 2012 (ml/min)	44.8(32.5–58.5)	42.1(32.1–57.9)	57.3(43.2–66.4)
ΔeGFR (ml/min)	-2.8(-7.9–1.4)	-2.9(-8.2–1.3)	-2.4(-7.8–1.3)
Blood glucose (mg/dl)	101.0(90.8–117.5)	100.0(89.0–114.5)	110.5(100.0–128.0)
LDL-C mg/dl	104.0(86.8–123.5)	104(91.3–125)	88.5(75.0–119.0)
HDL-C mg/dl	63.0(53.0–81.8)	62.0(53–83.3)	69.5(57.0–79.0)
LDL-C/HDL-C ratio	1.68(1.19–2.15)	1.7(1.2–2.1)	1.4(0.84–2.43)
Triglyceride	146.0(101.8–208.5)	153(106–220)	314.0(101.0–167.0)
Total ADPN at 2012 (μg/ml)	10.1(6.5–14.9)	9.5(6.3–14.5)	11.3(6.6–15.9)
HMW ADPN(μg/ml)	3.8(1.7–6.9)	3.6(1.8–6.8)	5.8(1.6–7.8)
MMW ADPN(μg/ml)	1.5(1.0–2.6)	1.5(1.1–2.6)	1.6(0.9–2.5)
LMW ADPN(μg/ml)	4.3(3.2–5.3)	4.3(3.2–5.3)	4.4(2.9–5.9)
HMW ADPN ratio (%)	40.6(29.1–48.1)	39.9(30.3–47.4)	46.7(24.6–52.8)
LMW ADPN ratio (%)	42.5(34.9–51.9)	43.9(34.8–51.7)	38.7(35.2–61.1)
Visceral fat area (cm^2^)	91.8(64.3–153.9)	82.3(60.1–133.4)	104.8(76.8–222.9)
Subcutaneous fat area (cm2)	96.2(57.3–131.4)	92.4(63.3–131.5)	15.1(46.9–142.1)
Therapeutic agents (Drug use, %)			
Immunosuppressive drugs			
Steroids	57(100%)	47(100%)	10(100%)
Antimetabolites	53(93%)	44(93%)	9(90%)
Calcineurin inhibitors	41(72%)	31(66%)	10(100%)*
Antihypertensive drugs			
ACEi/ARB	48(84%)	41(87%)	7(70%)
Antidiabetic drugs	27(47%)	22(47%)	5(50%)
Insulin	1(2%)	1(2%)	0(0%)
Oral antidiabetic drugs	8(14%)	8(17%)	0(0%)*
Statins	37(64%)	26(55%)	7(70%)
Cardiovascular disease	11(19%)	9(19%)	2(20%)
Angina pectoris	9(16%)	7(15%)	2(20%)
Aortic aneurysm	2(4%)	2(4%)	0(0%)
Cerebral infarction	(1)(2%)	(1)(2%)	0(0%)

Value are shown as the Median (IQR) *<0.05, **<0.01. Abbreviations: HLA: human leukocyte antigen, Tx: transplantation, eGFR: estimated-glomerular filtration rate, LDL-C: low-density lipoprotein cholesterol, HDL-C: high-density lipoprotein cholesterol, ADPN: adiponectin, ACEI: angiotensin converting enzyme inhibitor, ARB: angiotensin converting enzyme inhibitor. One patient is complicated by cerebral infarction and angina pectoris.

Comparisons between the genders are shown in Table 2. The post-transplant period was longer in males (p<0.05). LDL-C and the LDL-C/HDL-C ratio were higher in males (p<0.05 and p<0.01, respectively), while HDL-C was higher in females (p<0.05). Although no significant difference was noted in the serum ADPN level, it was slightly higher in females. SFA was higher in females (p<0.05).

**Table 2:** Characteristics of study subjects in gender.

Variable	Total	Male	Female
N	57	39	18
Age at transplantation, year	32.0(25.0–36.0)	33.0(25.0–36.0)	30.0(26.0–38.0)
HLA mismatches, median (range)			
HLA-A/B	2(0–4)	2(0–4)	2(0–4)
HLA-DR	1(0–2)	1(0–2)	0(0–2)
Time after Tx, month	257(188–333)	257(205–351)	219(163–307)*
Body mass index (BMI)	21.2(19.1–22.9)	21.3(19.3–23.8)	20.3(18.9–22.2)
Blood pressure, mmHg			
Systolic	126(120–132)	126(120–132)	123(112–128)
Diastolic	76(70–84)	76(70–84)	74(70–80)
eGFR at 2004 (ml/min)	55.2(43.5–68.6)	56.4(46.4–70.4)	50.1(40.5–59.3)
eGFR at 2008 (ml/min)	47.7(37.8–57.8)	48.0(38.9–60.6)	43.1(36.8–56.7)
eGFR at 2012 (ml/min)	44.8(32.5–58.5)	44.9(32.9–63.9)	41.8(31.0–56.3)
ΔeGFR(ml/min)	-2.8(-7.9–1.4)	-2.8(-5.5–2.7)	-2.4(-9.1–0.5)
Blood glucose (mg/dl)	101.0(90.8–117.5)	108.0(96.0–128.8)	93.0(84.0–100.0)
LDL-C mg/dl	104.0(86.8–123.5)	113.0(95.3–128.3)	90.5(82.0–104.0)*
HDL-C mg/dl	63.0(53.0–81.8)	59.0(52.0–71.3)	76.0(59.0–92.0)*
LDL-C/HDL-C ratio	1.7(1.19–2.15)	1.90(1.47–2.25)	1.23(1.00–1.63)**
Triglyceride	146.0(101.8–208.5)	146.0(99.8–216.0)	162.5(112.0–196.0)
Total ADPN at 2012 (μg/ml)	10.1(6.5–14.9)	8.8(5.9–14.3)	11.9(8.2–17.2)
HMW ADPN(μg/ml)	3.8(1.7–6.9)	3.3(1.4–6.5)	5.8(2.9–7.7)
MMW ADPN(μg/ml)	1.5(1.0–2.6)	1.5(0.9–2.4)	1.7(1.4–3.1)
LMW ADPN(μg/ml)	4.3(3.2–5.3)	4.3(3.2–5.0)	4.4(2.9–6.2)
HMW ADPN ratio(%)	40.6(29.1–48.1)	38.4(24.6–46.8)	45.7(39.6–48.5)
LMW ADPN ratio(%)	42.5(34.9–51.9)	45.1(37.9–55.5)	37.6(31.5–42.5)
Visceral fat area (cm^2^)	88.1(60.6–137.1)	106.6(66.3–165.9)	80.2(58.5–104.4)
Subcutaneous fat area (cm^2^)	94.9(61.9–131.7)	85.1(53.7–121.1)	118.5(88.7–141.4)*
Therapeutic agents (Drug use, %)			
Immunosuppressive drugs			
Steroids	57(100%)	39(100%)	18(100%)
Antimetabolites	54(95%)	37(94%)	17(94%)
Calcineurin inhibitors	41(72%)	26(67%)	15(83%)
Antihypertensive drugs			
ACEi/ARB	51(82%)	35(89%)	13(72%)
Antidiabetic drugs	27(48%)	19(49%)	8(44%)
Insulin	1(2%)	1(2.4%)	0(0%)
Oral antidiabetic drugs	8(13%)	6(15%)	2(11%)
Statins	33(58%)	22(56%)	11(61%)

Value are shown as the Median (IQR) *<0.05, **<0.01.

### Relationship between eGFR and ADPN in renal graft recipients

**Fig 1:**
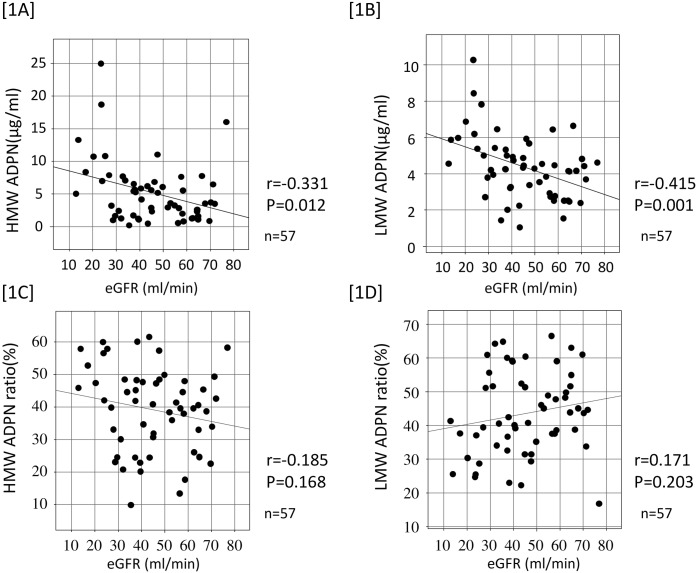
Correlation between serum adiponectin value and eGFR. Serum HMW and LMW ADPN level were negatively correlated with eGFR [1A] and [1B]. But HMW and LMW ADPN ratio were not significantly correlated with eGFR [1C] and [1D].

The relationship between renal graft recipients and serum ADPN levels is shown in Fig 1. HMW and LMW ADPN levels inversely correlated with eGFR (1A, 1B), whereas no correlation with eGFR was noted for either the HMW or LMW ADPN ratio (1C, 1D).

### Relationships between serum lipid markers and ADPN and fat areas in renal graft recipients

The relationships between serum lipid marker levels and fat areas in renal graft recipients are shown in Table 3. VFA positively correlated with LDL-C, the LDL-C/HDL-C ratio, and non HDL-C levels (r = 0.376, p = 0.004; r = 0.453, p<0.001; and r = 0.456, p<0.001; respectively), and inversely correlated with HDL-C (r = -0.285, p = 0.032). VFA inversely correlated with HMW and LMW ADPN levels (r = -0.400, p = 0.002 and r = -0.296, p = 0.025, respectively), as did SFA (r = -0.319, p = 0.015 and r = -0.386, p = 0.003, respectively). VFA also inversely correlated with the HMW ADPN ratio, whereas it positively correlated with the LMW ADPN ratio (r = -0.444, p<0.001 and r = 0.467, p<0.001, respectively). SFA did not correlate with either ratio.

**Table 3:** The correlation between visceral, subcutaneous fat area and each lipid marker.

	Visceral fat area		Subcutaneous fat area	
	r	p value	r	p value
LDL-C	0.376	0.004	0.225	0.093
HDL-C	-0.285	0.032	-0.149	0.269
LDL-C/HDL-C ratio	0.453	<0.001	0.222	0.097
non HDL-C	0.456	<0.001	0.229	0.085
HMW ADPN	-0.400	0.002	-0.319	0.015
HMW ADPN ratio (%)	-0.444	<0.001	-0.152	0.258
LMW ADPN	-0.296	0.025	-0.386	0.003
LMW ADPN ratio (%)	0.467	<0.001	0.122	0.368

HMW ADPN: High molecular weight adiponectin, LMW ADPN: Low molecular weight adiponectin.

**Fig 2:**
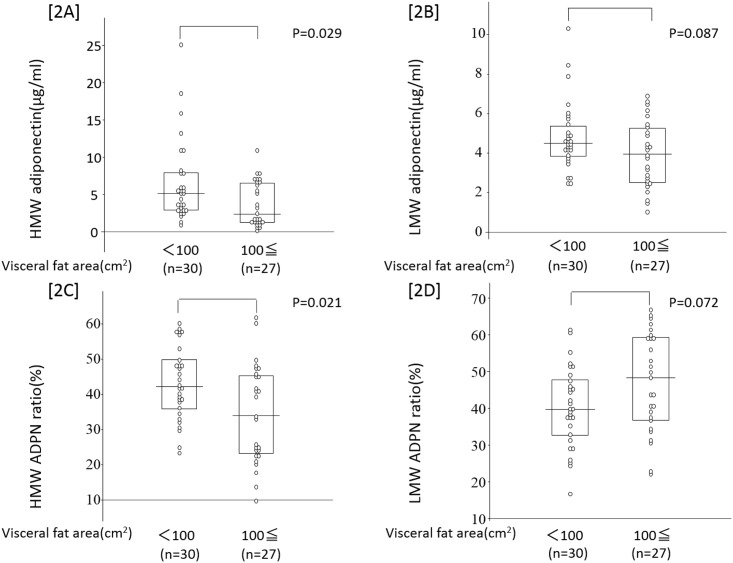
A comparison of serum adiponectin value in obese patients and non-obese patients. Obese patients were stratified by visceral fat accumulation (VFA cutoff value; 100cm2. Serum HMW ADPN level and HMW ADPN ratio were lower in obese patients [2A] and [2C]. LMW ADPN ratio was higher in obese patients [2D]. There was no significant difference in LMW adiponectin level between two groups [2B].

Our subjects were divided into those who were and were not obese based on the definition of obesity in Japanese individuals (100 cm^2^ or higher VFA), and ADPN levels and ratios were compared. HMW ADPN and its ratio were lower in obese subjects (p<0.029 and p<0.021, respectively), whereas no significant difference was noted in LMW ADPN levels or its ratio (Fig 2).

The factors influencing HMW ADPN levels and its ratio were investigated using a multiple regression analysis (Table 4). The models regarding HMW ADPN and its ratio as a response variable were designated as Models 1 and 2, respectively. In Model 1, eGFR and VFA were significant HMW ADPN level-lowering factors, and age at transplantation was detected as an increasing factor. In Model 2, VFA was a significant HMW ADPN ratio-reducing factor.

**Table 4:** Factors influenced of factor on HMW ADPN and HMW ADPN ratio in renal transplant subjects.

Model 1					
Objective variable: HMW ADPN					
	β	SE	Std β	t	p
(Constant)	8.118	2.202			
eGFR	-0.09	0.033	-0.318	2.738	0.008
Visceral fat area	-0.019	0.008	-0.308	2.379	0.021
Age at transplantation	0.124	0.056	0.267	2.237	0.029
DM	-2.494	1.439	-0.225	1.732	0.089
Model 2					
Objective variable: HMW ADPN ratio					
	β	SE	Std β	t	p
(Constant)	47.45	2.807			
DM	-6.699	4.057	-0.22	1.652	0.104
Visceral fat area	-0.059	0.023	-0.345	2.587	0.012

Explanatory variable: cardiovascular disease, DM, age at transplantation, gender, cadaver/living, statin use, ARB use, eGFR, HDL-C, LDL-C, LDL/HDL-C, non HDL-C, duration after transplantation, visceral fat area, subcutaneous fat area.

### Relationships between the development of PTDM and ADPN and fat areas

**Fig 3:**
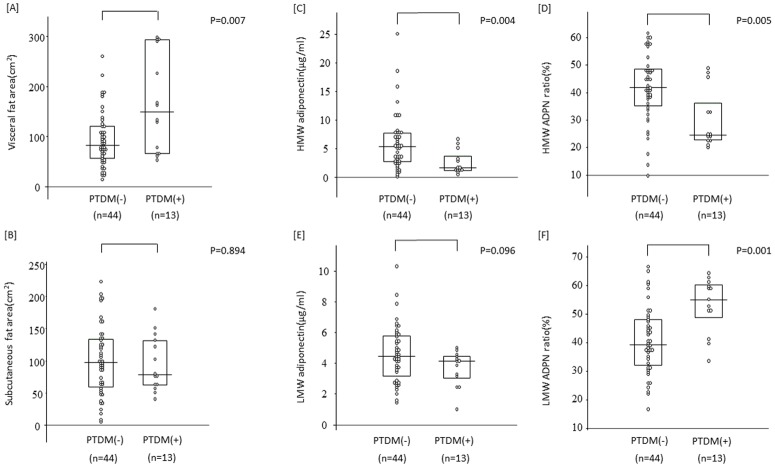
The association of visceral fat area and serum adiponectin value between PTDM and non-PTDM patients. Visceral fat area were higher in PTDM patients [A]. There were no significant difference in subcutaneous fat area [B]. Serum HMW ADPN level and HMW ADPN ratio were lower in PTDM patients [C] and [D]. LMW ADPN ratio was higher in PTDM patients [F]. There was no significant difference in LMW ADPN level between two groups [E].

The relationships between the development of PTDM with serum ADPN levels and fat areas are shown in Fig 3. VFA was higher in PTDM than in non-PTDM patients ([A]: p = 0.007), whereas no significant difference was noted in SFA ([B]: p = 0.984). The HMW ADPN level and its ratio were lower in PTDM patients ([C]: p = 0.004 and [D]: p = 0.005, respectively), while the LMW ADPN ratio was higher ([F]: p = 0.001). The factors involved in the development of PTDM were investigated by a multiple regression analysis, with increases in VFA and the LMW ADPN ratio being identified as significant increasing factors (Table 5).

**Table 5:** The influenced factor on post transplant DM in renal transplant subjects.

	β	SE	z value	p	Odds ratio
(Constant)	-8.517	3.384			
Visceral fat area	0.016	0.008	2.014	0.044	1.016
LMW ADPN ratio	0.132	0.055	2.39	0.017	1.142
Donor cadaveric	-2.933	1.613	1.818	0.069	0.053
Age at transplantation	0.098	0.061	1.603	0.108	1.103
Statin use	-2.153	1.154	1.865	0.062	0.116

Explanatory variable: age at transplantation, gender, cadaver/living, CIN, statin use, ARB use, eGFR, HDL-C, LDL-C, LDL/HDL-C, non HDL-C, duration after transplantation, visceral fat area, subcutaneous fat area, HMW ADPN ratio, LMW ADPN ratio.

### Relationships between the development of CVD and ADPN and fat areas

**Fig 4:**
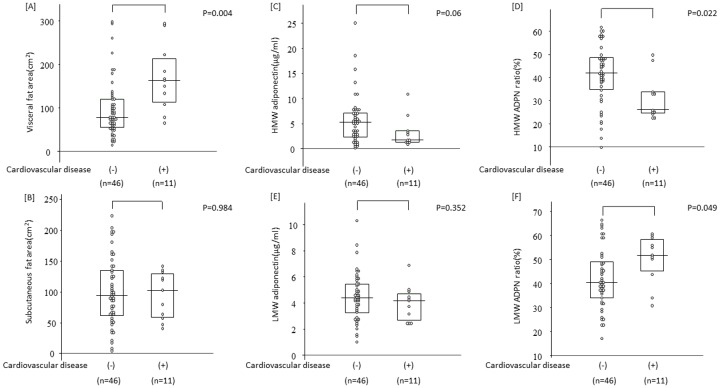
The association of visceral fat area and serum adiponectin value between CVD and non-CVD patients. Visceral fat area were higher in CVD patients [A]. There were no significant difference in subcutaneous fat area between two groups [B]. HMW ADPN levels and HMW adiponectin ratio were lower in CVD patients [C] and [D]. LMW ADPN ratio was higher in CVD patients [F]. There was no significant difference in LMW ADPN level between two groups [E].

The relationships between the development of CVD and serum ADPN levels and fat areas are shown in Fig 4. Among 11 subjects who developed CVD after transplantation, 5 developed angina pectoris, 3 ischemic heart disease-induced heart failure, 2 aortic aneurysm, 1 aortic dissection, and 2 cerebral infarction. VFA was higher in CVD patients ([A]: p = 0.004), whereas no significant difference was noted in SFA ([B]: p = 0.984), and the HMW and LMW ADPN ratios were also higher ([D]: p = 0.022 and [F]: p = 0.049, respectively). The factors involved in the development of CVD were investigated using a multiple regression analysis. An increase in the HMW ADPN ratio and treatments with statins were reducing factors, while an increase in LDL-C was an increasing factor (Table 6).

**Table 6:** The influenced factor on cardiovascular disease in renal transplant subjects.

	β	SE	z value	p	Odds ratio
(Constant)	-0.495	3.028			
HMW ADPN ratio	-0.159	0.072	2.225	0.026	0.853
Statin use	-3.554	1.502	2.367	0.018	0.029
LDL-cholesterol	0.075	0.029	2.586	0.009	1.078
Subcutaneous fat area	-0.03	0.018	1.706	0.088	0.97

Explanatory variable: age at transplantation, gender, cadaver/living, CIN, statin use, ARB use, eGFR, HDL-C, LDL-C, LDL/HDL-C, non HDL-C, duration after transplantation, visceral fat area, subcutaneous fat area, HMW ADPN ratio, LMW ADPN ratio

## Discussion

The present study obtained 3 main results. Serum HMW and LMW ADPN levels inversely correlated with eGFR in renal transplant patients, whereas their ratios did not, and the serum HMW ADPN level and its ratio were associated with VFA. Furthermore, the development of CVD in renal transplant patients was associated with VFA and the HMW ADPN ratio. In addition, the development of PTDM was associated with VFA and the LMW ADPN ratio.

A large number of studies have examined the relationships between serum total and HMW ADPN levels and renal function: eGFR and serum total and HMW ADPN levels inversely correlated in CKD patients, and improved renal function was associated with reductions in plasma ADPN levels [13, 14]. A decrease in plasma ADPN levels after kidney transplantation has also been reported [15, 16]. The reasons for these findings include the expression of renal graft function and an improved inflammatory environment [17]. However, fewer studies have investigated the relationship between the serum ADPN fraction and renal graft function. In the present study, renal graft function and HMW and LMW ADPN levels inversely correlated, whereas no correlation was noted in either of their ratios. Regarding the applicability of the serum HMW ADPN ratio to evaluations of metabolic syndrome, the usefulness of comparisons with serum total and HMW ADPN levels has been reported [18, 19]. However, LMW ADPN levels and its ratio in renal transplant patients currently remain unknown. Since circulating ADPN levels including HMW and LMW ADPN is influenced by renal function, our results suggest that evaluating the ADPN ratio is useful for investigating the qualitative changes of ADPN in CKD and renal transplant patients.

The relationship between obesity (VFA, SFA) and ADPN has been extensively examined. A decrease in serum ADPN levels in obese patients [20] and an inverse correlation between serum ADPN levels and VFA has been reported, whereas a correlation has not been observed with SFA [10]. Previous studies also showed inverse correlations between waist circumference and LMW ADPN and serum IL-6 levels [21], and also between fat areas and LMW ADPN levels [22, 23]. In the present study, VFA was inversely correlated with serum HMW and LMW ADPN levels and HMW ADPN ratio. Furthermore, VFA was positively with the LMW-ADPN ratio, but no significant correlation was noted between SFA and the ADPN ratios. These findings suggest that evaluations of VFA, not SFA are important for determining the relationship between serum ADPN and obesity in renal transplant patients. Since it has not yet been established whether waist circumference and BMI accurately reflect VFA, VFA and SFA are currently evaluated separately. Therefore, it is important to distinguish and measure individual fat areas using abdominal CT.

Blood ADPN levels are known to be significantly lower in coronary arterial disease patients than in an age- and obesity grade-matched control group [24]. The prevalence of coronary arterial disease in males was also found to be 2-fold higher in a group with a blood ADPN level lower than 4 μg/ml than in a group with a level of 7 μg/mL or higher and this was independent of other risk factors [9]. Regarding the relationship between HMW ADPN and cardiovascular complications, Sato et al. reported that HMW ADPN levels and its ratio were significantly lower in coronary arterial disease patients than in a normal subject group [25]. Furthermore, relationships have been found between ADPN and arteriosclerosis regardless of sex [26] and between an increase in VFA and coronary arterial calcification in male CKD patients [27]. In this study, patients with CVD showed larger VFA, lower HMW ADPN ratio and higher LMW ADPN ratio, but not in SFA. Even in small group, our present study also identified an increase in the HMW ADPN ratio as a factor reducing the incidence of CVD. These findings demonstrate that CVD in renal transplant patients is associated with ADPN fraction and VFA.

In spite of ADPN being secreted by adipocytes, its blood level is low in obese individuals, and is even lower in patients with type II diabetes [28]. Serum total and HMW ADPN levels are known to be lower in diabetes patients than in healthy subjects [29], and inverse correlations have been noted between serum ADPN levels and VFA with insulin resistance [30, 31]. Moreover, a relationship has been reported between diabetes and LMW ADPN levels. Serum LMW ADPN levels were found to be lower in type II diabetes patients than in non-obese patients, and an inverse correlation was detected between waist circumference and serum LMW ADPN levels [20]. Furthermore, LMW ADPN levels were found to be lower in elderly patients with type II diabetes than in non-diabetic patients [32]. In our analysis, we found not only HMW, but also LMW ADPN as factors influencing the development of PTDM.

As for pathogenic difference of ADPN fractions, previous study demonstrated that LMW and MMW ADPN passed through the blood-brain barrier and activated AMP kinase through the hypothalamic AdipoR1 receptor, which increased food intake, reduced energy consumption, and resulted in fat accumulation [33]. These findings showed that, regarding body weight gain and loss, the functions of LMW and MMW ADPN are opposite to those of HMW ADPN. The increase observed in the LMW ADPN ratio in the present study may have resulted from qualitative changes in adipocytes induced by an obesity-associated chronic inflammatory state. However, the function of LMW ADPN remains unclear; therefore, further investigations are warranted.

### Limitations

There were the following limitations to this study: 1) the small number of subjects is the study at risk for bias due to the population studied (Japanese population). Hence, our findings may not be able to generalize to other populations. 2) it was retrospective in nature. 3) the use of immunosuppressive drugs and statins was not uniform. Therefore, a prospective study is needed in order to investigate relationships with changes in ADPN. 4) we could not evaluate the risk of smoking in this cohort. It may be another important limitation to compare with the reports from other countries given that a significant percentage of kidney transplant recipients smoke.

## Conclusion

We herein demonstrated that VFA and GFR are associated with serum HMW and LMW ADPN levels even in Japanese renal transplant patients. Otherwise, the ADPN ratio is more important for developing CVA and PTMD. In this notion, the higher HMW-ADPN ratio and statin treatment were identified as reducing factors of the development of CVD. On the other hand, the higher LMW-ADPN ratio and the VFA were aggravating factors of PTDM.

## Supporting Information

S1 Fig Measurement methods of fat area. The abdominal wall was traced to separate subcutaneous and visceral fat for the measurement of the CT values of 10-mm thick and excised areas (-50 ~ -150) in the umbilicus. If the kidney and ilium were included in the umbilical region, the data should be obtained from the regions avoiding them as much as possible.
(TIF)

S2 Fig Stratified evaluation of high- and low-molecular-weight ADPN levels by visceral fat area. The visceral fat area was divided into quartiles to evaluate serum adiponectin levels. As a result, the high-, but not low-, molecular-weight adiponectin level decreased as the visceral fat area increased.
(TIF)

S3 Fig Stratified evaluation of high- and low-molecular-weight ADPN ratio by visceral fat area. The visceral fat area was divided into quartiles to evaluate serum adiponectin fractions. As a result, the high-molecular-weight adiponectin fraction decreased, while the low-molecular-weight adiponectin fraction increased, as the visceral fat area increased.
(TIF)
