# Supplementary figures and images for: Adiponectin Fractions Influence the Development of Posttransplant Diabetes Mellitus and Cardiovascular Disease in Japanese Renal Transplant Recipients
Source: PLoS One. 2016 Oct 5;11(10):e0163899. doi: 10.1371/journal.pone.0163899 (PMC5051963; doi:10.1371/journal.pone.0163899)

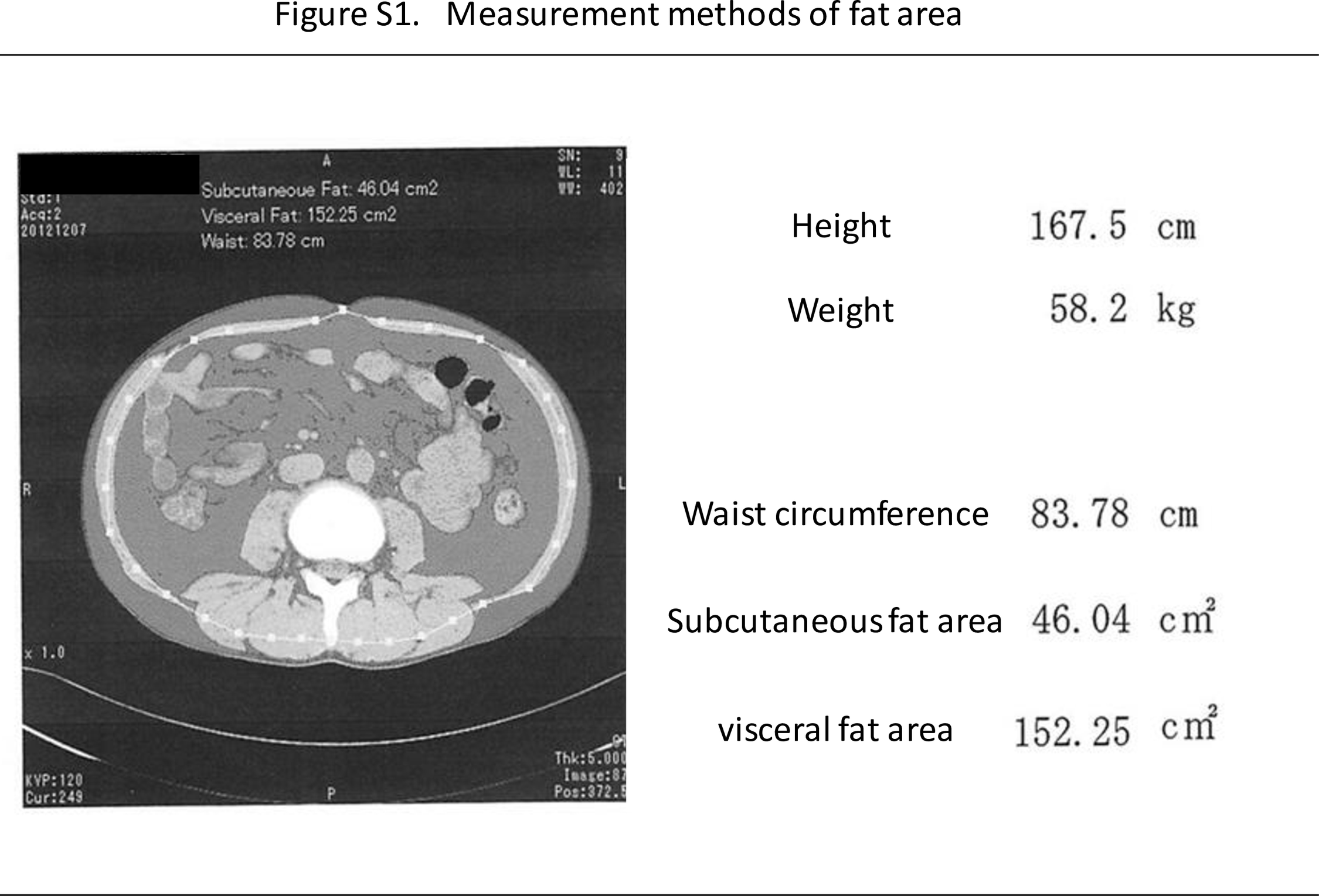

Supplement: S1 Fig — The abdominal wall was traced to separate subcutaneous and visceral fat for the measurement of the CT values of 10-mm thick and excised areas (-50 ~ -150) in the umbilicus. If the kidney and ilium were included in the umbilical region, the data should be obtained from the regions avoiding them as much as possible. (TIF) [file pone.0163899.s001.tif]

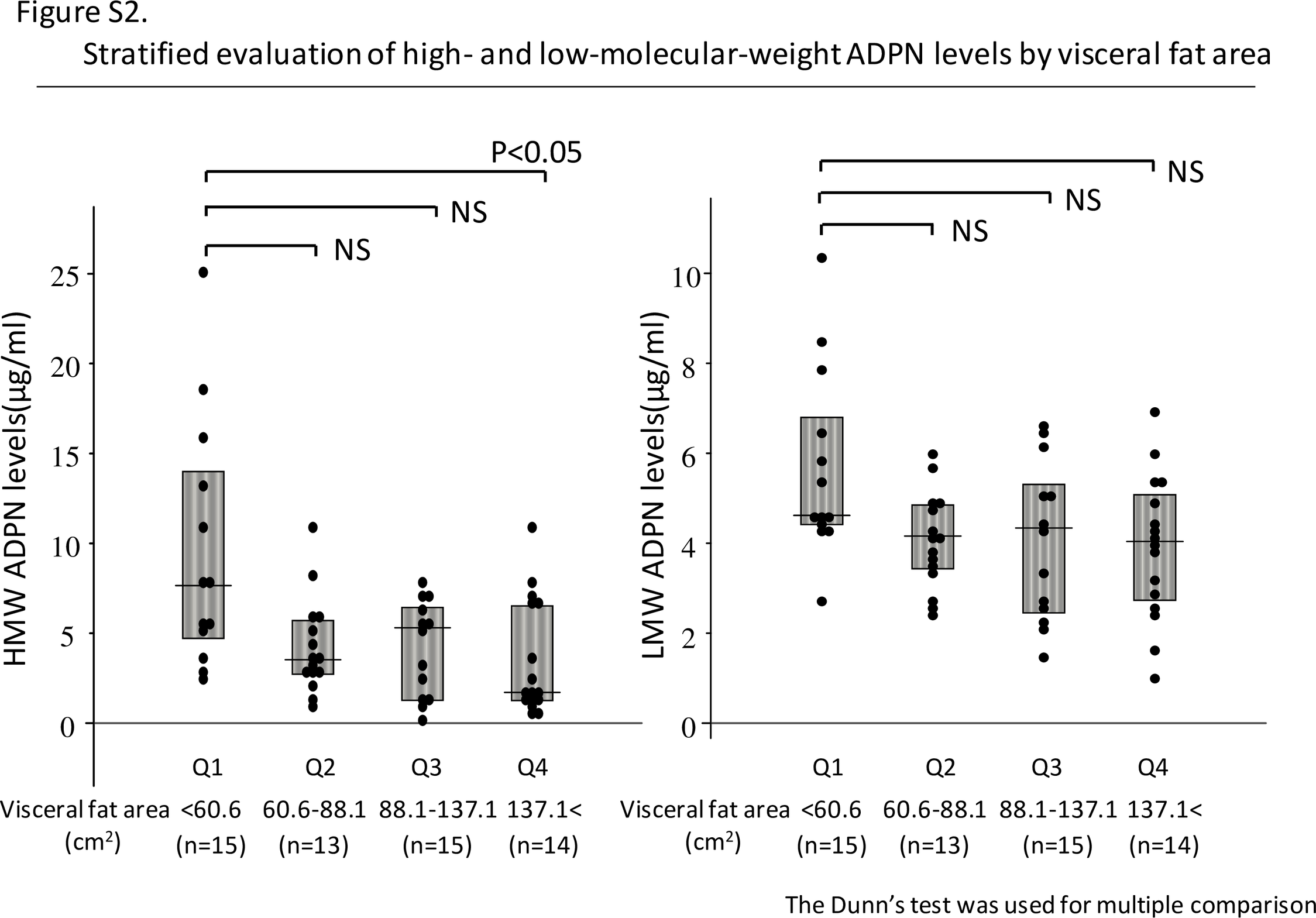

Supplement: S2 Fig — The visceral fat area was divided into quartiles to evaluate serum adiponectin levels. As a result, the high-, but not low-, molecular-weight adiponectin level decreased as the visceral fat area increased. (TIF) [file pone.0163899.s002.tif]

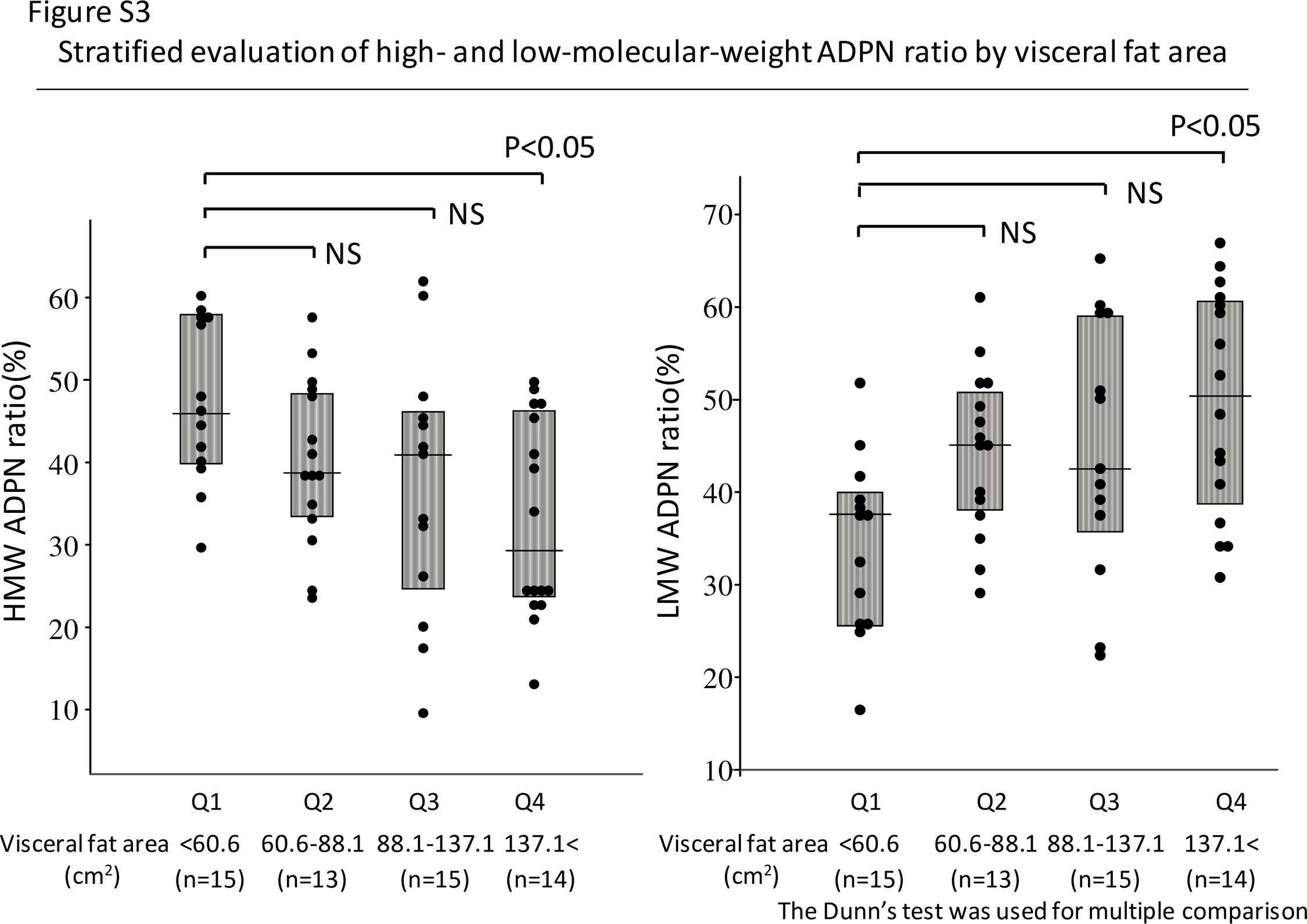

Supplement: S3 Fig — The visceral fat area was divided into quartiles to evaluate serum adiponectin fractions. As a result, the high-molecular-weight adiponectin fraction decreased, while the low-molecular-weight adiponectin fraction increased, as the visceral fat area increased. (TIF) [file pone.0163899.s003.tif]
